# Does Cyclic ADP-Ribose (cADPR) Activate the Non-selective Cation Channel TRPM2?

**DOI:** 10.3389/fimmu.2020.02018

**Published:** 2020-08-11

**Authors:** Ralf Fliegert, Winnie M. Riekehr, Andreas H. Guse

**Affiliations:** The Calcium Signalling Group, Department of Biochemistry and Molecular Cell Biology, University Medical Center Hamburg-Eppendorf, Hamburg, Germany

**Keywords:** TRPM2, ion channel, calcium, signal transduction, cADPR

## Abstract

TRPM2 is a non-selective, Ca^2+^-permeable cation channel widely expressed in immune cells. It is firmly established that the channel can be activated by intracellular adenosine 5′-diphosphoribose (ADPR). Until recent cryo-EM structures have exhibited an additional nucleotide binding site in the N-terminus of the channel, this activation was thought to occur via binding to a C-terminal domain of the channel that is highly homologous to the ADPR pyrophosphatase NudT9. Over the years it has been controversially discussed whether the Ca^2+^ mobilizing second messenger cyclic ADP ribose (cADPR) might also directly activate Ca^2+^ entry via TRPM2. Here we will review the status of this discussion.

## Introduction

TRPM2 is a non-selective, Ca^2+^-permeable cation channel expressed in immune cells like monocytes (1, 2), macrophages (3–5), neutrophils (6–9), dendritic cells (10) and effector T cells (11). The channel plays a role in the inflammatory response by modulating differentiation (10), cell migration and chemotaxis (7, 10, 12), cytokine (11) and chemokine secretion (1) and is regulated in a complex manner integrating inputs from the physical environment of the cell like temperature (13) and pH (14) with intracellular second messengers like Ca^2+^ (15, 16) and adenine nucleotides. Since cloning of TRPM2 over 20 years ago (17), a number of adenine nucleotides have been proposed to affect TRPM2. While ADPR and 2′-deoxy-ADPR (18) are firmly established as TRPM2 agonists, the roles of NAADP and cADPR in activation of the channel remain controversial. In this review we want to summarize the literature regarding the role cADPR with an emphasis on recent (structural) data.

In 2001 Perraud et al. found that the cytosolic C-terminus of TRPM2 (at that time known as LTRPC2) contains a Nudix box motif (19). This sequence motif is known from a huge superfamily of proteins, many of them pyrophosphorylases that hydrolyse “nucleoside diphosphates linked to a residue X” (hence the name NudiX) [reviewed in Srouji et al. (20)]. Nudix pyrophosphorylases differ largely with regard to substrate specificity with some of them having only a single substrate while others hydrolyse a broad range of dinucleotides. By sequence analysis Perraud et al. discovered the gene for an enzyme, now known as NudT9, that exhibits 50% sequence homology to TRPM2. By testing a number of potential substrates, they identified adenosine 5′-diphosphoribose (ADPR) as its substrate (19). ADPR is a cellular nucleotide that can arise from hydrolysis of NAD by the NAD glycohydrolase CD38 (21, 22) or may be cleaved from poly-ADP-ribosylated or mono-ADP-ribosylated proteins (23). Perraud et al. also demonstrated that ADPR can activate TRPM2 in a Ca^2+^-dependent manner supposedly by binding to its C-terminal NudT9 homology domain (19).

Kolisek et al. later found that cyclic ADP ribose (cADPR), another metabolite of NAD, is also able to activate TRPM2 (24). cADPR is a second messenger in a number of different cell types [reviewed in Lee (25)] including cells of the immune system like T cells (26) and neutrophils (27) that mobilizes Ca^2+^ from intracellular stores (26). The cellular target for cADPR remains still elusive. While photoaffinity labeling with a cADPR analog in sea urchins pointed to a receptor with a molecular weight of 100–140 kDa which has so far escaped identification (28), most data for higher animals indicate that Ca^2+^ release by cADPR involves ryanodine receptors type 2 (29) or type 3 (30). This might be by displacement of FKBP12.6 from the ryanodine receptors resulting in an increased open probability (31, 32) or by indirect mechanisms like increasing store loading by stimulating activity of the ER Ca^2+^ pump SERCA (33–35).

In addition to releasing Ca^2+^ from intracellular stores, it has also been shown that cADPR can trigger Ca^2+^ entry via the plasma membrane. In Jurkat T cells microinjection of cADPR activates Ca^2+^ entry over the plasma membrane (36) and in neutrophiles the cADPR antagonist 8-Br-cADPR inhibits Ca^2+^-influx in response to the chemotactic peptide fMLP (27). So far it is unclear whether this Ca^2+^ influx works via activation of capacitative Ca^2+^ entry via the STIM/Orai system (37) or involves additional Ca^2+^ channels directly activated by cADPR. These findings made the observation that cADPR might activate TRPM2 especially interesting.

Activation of TRPM2 by cADPR requires exceedingly high concentrations (EC_50_ 700 μM) of cADPR and even at 3 mM cADPR in the patch pipette the current was only about 5% of the current evoked by ADPR in low micromolar concentrations (24). Cellular concentrations of cADPR determined in the past by us and others using either HPLC (26, 38) or an enzymatic cycling assay (39–41) are significantly lower. This makes it highly unlikely that cADPR alone can contribute to Ca^2+^ entry by activation of TRPM2. On the other hand did cADPR shift the concentration-response for ADPR by two orders of magnitude from an EC_50_ of 12 μM in the absence of cADPR to 90 nM in the presence of 10 μM cADPR, resulting in the hypothesis that cADPR and ADPR may activate TRPM2 synergistically (24).

## Potential Binding Site of cADPR at TRPM2

**FIGURE 1:**
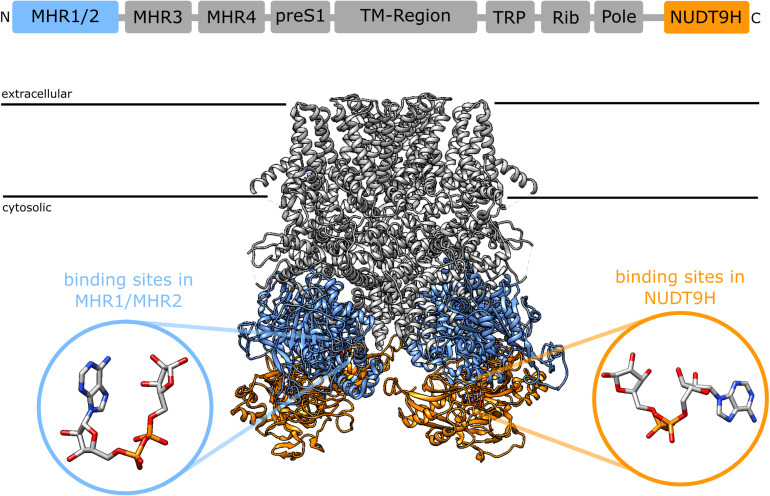
Domain structure of TRPM2 [after (46)] and location of the two nucleotide binding sites in the cryoEM structure of human TRPM2 [pdb: 6PUS, (48)]: the recently identified N-terminal binding site between the MHR1 and MHR2 domains (blue) and the established C-terminal binding site in the NudT9H domain. The insets show the different conformation of ADPR in the binding sites as determined from the cryoEM structure, the ADPR in the N-terminal binding site assumes a horseshoe like conformation whereas the ADPR in in the NudT9H domain has a more elongated conformation. The depicted ADPR molecules have been reoriented relative to the TRPM2 structure to better illustrate the difference in conformation between the two binding sites. In a structure that has been solved in the presence of 8-Br-cADPR, 8-Br-cADPR occupies the N-terminal binding site between MHR1/MHR2 (48).

A synergism between cADPR and ADPR raises the question of the binding site. 8-Br-cADPR, an antagonist of cADPR (42) inhibited activation of TRPM2 by cADPR but not by ADPR, whereas AMP, one of the products of the enzyme NudT9, inhibited activation by ADPR but not by cADPR, indicating that ADPR and cADPR do not act via the same binding site. Since AMP affects activation by ADPR it seems that ADPR binds to the NudT9H domain, whereas cADPR would bind to a distinct site for which it competes with 8-Br-cADPR. First indications of a secondary nucleotide binding site came from work on TRPM2 from the sea anemone *Nematostella vectensis* (nvTRPM2). nvTRPM2 also features a Nudix domain, but Kühn et al. showed that removal of this NudT9H domain does not interfere with gating of the channel by ADPR, but that the domain is catalytically active and breaks down ADPR (43). This led them to propose that the ADPR binding site for nvTRPM2 is separate from the NudT9H domain. Later they showed that the NudT9H domain of nvTRPM2 while not required for activation by ADPR can contribute to gating. While in nvTRPM2, activation by ADPR is not affected by removal of the NudT9H domain, activation by inosine 5′-diphosphoribose (IDPR) is abrogated. This shows that the second binding sites can modulate channel activity and exhibits a different agonist selectivity (44). During the last year a number of cryo-EM structures of TRPM2 from different species became available (45–48). One especially interesting finding from the studies by Huang et al. was the identification of additional nucleotide binding sites in TRPM2 from zebra fish (drTRPM2) (47) and humans (48). Located between the first two melastatin homology regions (MHR1/MHR2) in the cytosolic N-terminus of the channel they observed an ADPR molecule in a horseshoe-like conformation that resembles the conformation of cADPR in both zebra fish and human TRPM2 (Figure 1). In human TRPM2 they were able to resolve an additional ADPR molecule in the NudT9H domain which exhibited in contrast to the horse-shoe-like ADPR in the MHR1/MHR2 binding site an elongated confirmation (Figure 1). They also solved a structure of TRPM2 in an inhibited state with the cADPR antagonist 8-Br-cADPR in the presence of Ca^2+^. The functional role of these distinct nucleotide binding sites remains controversial. While Huang et al. observed a loss of activity when mutating the MHR1/MHR2 binding site as well as when removing the NudT9H domain in both zebra fish (47) as well as human TRPM2 (48), Wang et al. did neither observe any ADPR related electron density in the MHR1/MHR2 domain nor did they see an effect of the mutation of this site (46).

## Temperature Dependency of cADPR Mediated TRPM2 Activation

Togashi et al. first noticed that TRPM2 can be activated by heat above a threshold temperature of 35°C with currents increasing up to 42°C (13). While ADPR activated TRPM2 already at 25°C, the currents were largely enhanced when the temperature was increased to 35°C and above. In contrast to Kolisek et al. Togashi et al. did not observe any cADPR evoked currents at 25°C but found that 100 μM cADPR in the pipette largely enhanced TRPM2 currents evoked by heat. This effect was absent in cells expressing a TRPM2 variant lacking the NudT9H domain (13).

Due to its labile N1-glycosidic bond cADPR is prone to hydrolysis to ADPR. At room temperature and under slightly acidic conditions its half-life is 10 days which decreases to 24 h at 37°C (49). Even frozen solutions of cADPR have been shown to slowly degrade to ADPR at -20°C (50). cADPR is also subject to enzymatic hydrolysis to ADPR by CD38 and CD157/Bst-1 which besides NAD glycohydrolase and ADP-ribosyl cyclase activity also exhibit cADPR hydrolase activity (51–53). Hydrolysis of the pyrophosphate in cADPR by an Mn^2+^-dependent ADP-ribose/CDP-alcohol pyrophosphatase yields N^1^-(5-phosphoribosyl)-AMP resulting in breakdown of cADPR without production of ADPR (54). Increasing temperature accelerates chemical and enzymatic turnover but due to the rapid kinetics it appears unlikely that increased hydrolysis of cADPR to ADPR is responsible for the results observed by Togashi et al. In addition Yu et al. demonstrated that wildtype HEK293 cells do not express CD38 or CD157/Bst-1 nor do they show turnover of cADPR to ADPR over the time course of a typical patch-clamp experiment (55).

## Contamination of Commercial cADPR Preparations

A complicating factor in interpreting the results from Kolisek et al. and Togashi et al. is, that commercial preparations of cADPR from one of the major suppliers are often partially degraded and contain significant amounts of ADPR. Heiner et al. noticed high currents when infusing cADPR into human neutrophils which prompted them to check their solutions for ADPR contaminations by HPLC (56). They found that even freshly prepared solutions from several batches of commercial cADPR, contained roughly 25% ADPR. When they incubated the contaminated cADPR with nucleotide pyrophosphatase thereby fully converting ADPR to AMP and ribose 5-phosphate, the ADPR-free cADPR did no longer evoke TRPM2 currents in the granulocytes (56). When using a commercial preparation of cADPR Tóth et al. also observed activation of TRPM2 by “cADPR” in inside-out patches from *Xenopus oocytes*, but analysis of the composition of the “cADPR” preparation by thin layer chromatography showed that in addition to cADPR it contained roughly 20% ADPR (57). Selective hydrolysis of ADPR by nucleotide pyrophosphatase, without degradation of cADPR (58), resulted in a complete loss of TRPM2 activation. Both groups noticed that the loss in channel activation was not due to inhibition by AMP as addition of the same amount of AMP to ADPR did not affect activation of the channel by ADPR (56, 57). Interestingly, in contrast to previous reports that showed inhibition by AMP with an IC_50_ of 70 μM (24) and later of 10 μM (8) Tóth et al. didn’t observe any inhibition of human TRPM2 expressed in Xenopus oocytes by AMP up to 200 μM (57).

Like for cADPR commercial preparations also 8-Br-cADPR often contains significant amounts of 8-Br-ADPR (>20%). The observation that 8-Br-ADPR is a low affinity competitive antagonist for ADPR on TRPM2 (7) [IC_50_ ∼300 μM (18)] further complicates interpretation of reports of selective inhibition of cADPR-mediated activation of TRPM2 (24). One conceivable explanation for these results might be that the administration of an excess of 8-Br-ADPR (from the 8-Br-cADPR) to a small amount of ADPR (as a contaminant in cADPR) is effectively preventing channel activation, while it has no effect on activation of TRPM2 by 100 μM ADPR. This could also explain how 8-Br-cADPR exerts its effects on H_2_O_2_-mediated activation of TRPM2 (24). Interestingly it has been shown recently that a variant of nvTRPM2 lacking the NudT9H domain can be activated by 8-Br-ADPR acting as a low affinity partial agonist (59), indicating that 8-Br-ADPR may bind to the N-terminal nucleotide binding domain of TRPM2. This and the amount of 8-Br-ADPR in commercial preparations of 8-Br-cADPR raises the question whether the resolution of the current cryo-EM structures is sufficient to exclude the possibility that the nucleotide observed in the N-terminal binding site in the pdb structure 6PUU (48) is not 8-Br-cADPR but 8-Br-ADPR in a horseshoe-like conformation.

Interestingly a relatively recent paper by Yu et al. again seems to demonstrate activation of human TRPM2 overexpressed in HEK cells by cADPR (55). The concentration-response curve for cADPR was shifted to the right with an EC_50_ of 250 μM compared to 40 μM for ADPR. In stark contrast to what has been observed by Kolisek et al. (24) the maximal currents for ADPR and cADPR were similar (55). They tried to account for the problems with ADPR contaminations described above by using cADPR they either synthesized themselves or purified from commercial cADPR and demonstrated purity by mass spectrometry. Although the previous data by Kolisek et al. indicated that cADPR binds to a different site than ADPR they assumed binding to the NUDT9H domain which they confirmed by showing the binding to the isolated NUDT9H domain using surface plasmon resonance. Using molecular dynamics simulation they identified a number of residues involved in binding to cADPR and ADPR. Mutations of some of these residues exhibited differential effects on channel activation by either ligand (55). It is really interesting to see, that even more than 10 years after Heiner et al. (56) and Tóth et al. (57) convincingly demonstrated that removal of contaminating ADPR prevents activation of TRPM2 by commercial cADPR, the idea that cADPR could affect TRPM2 still lingers on.

To avoid misleading results in the future, we consider it of utmost importance to always keep in mind both, the possibility that commercial preparations of cADPR can contain significant amounts of ADPR (even despite the advertised purity) and the limited stability of cADPR in solution, even when frozen. When working with cADPR we would therefore highly recommend to (i) purify commercial preparations before use, and (ii) to test for degradation of cADPR in solution routinely by using a suitable HPLC system.

## Author Contributions

All authors listed have made a substantial, direct and intellectual contribution to the work, and approved it for publication.

## Conflict of Interest

The authors declare that the research was conducted in the absence of any commercial or financial relationships that could be construed as a potential conflict of interest.
